# Critical Care Dilemma: Complex Congenital Pulmonary Airway Malformation With Pulmonary Hypertension in a Neonate

**DOI:** 10.7759/cureus.85279

**Published:** 2025-06-03

**Authors:** Sajjad M AlKadhem, Ali T Alattas, Hadeel AlJubab, Abdulwahhab S AlJubab

**Affiliations:** 1 Pediatric Intensive Care Unit, King Fahad Medical City, Riyadh, SAU; 2 Pediatric Surgery, King Fahad Medical City, Riyadh, SAU

**Keywords:** congenital pulmonary airway malformation, high-frequency oscillatory ventilation, hypoxic-ischemic injury, multidisciplinary management, neonatal intensive care, pediatric surgery, pneumothorax, prenatal diagnosis, pulmonary hypertension, ventilator-associated pneumonia

## Abstract

Congenital pulmonary airway malformations (CPAMs) are the most prevalent congenital lung lesions, yet their postnatal trajectory remains heterogeneous. Although many antenatally detected macrocystic lesions remain clinically silent, rapid postnatal enlargement can precipitate tension physiology, air-leak syndromes, and secondary pulmonary hypertension - events that mandate urgent departure from elective surgical timetables. We report a term female infant with a right-sided type 1 CPAM diagnosed by 20-week ultrasound. Despite an initial asymptomatic course, the lesion expanded abruptly at four weeks of age, producing severe hyperinflation, bilateral tension pneumothoraces, ventilator dependence, and refractory pulmonary hypertension. Optimization with high-frequency oscillatory ventilation, inhaled nitric oxide, dual oral vasodilator therapy, and chest-tube decompression facilitated physiological stabilization. Definitive management proceeded in two stages: cyst decompression and wedge resection at six weeks, followed by completion of middle lobectomy at 12 weeks. Postoperative complications - including a transient pulmonary hypertensive crisis, *Stenotrophomonas maltophilia* ventilator-associated pneumonia, and hypoxic-ischemic seizures - were managed successfully with targeted pharmacologic and supportive measures. This case underscores three practice points: (1) ostensibly quiescent CPAMs warrant vigilant surveillance for early indicators of mass-effect decompensation; (2) short, deliberate delays in definitive resection can be justified to optimize respiratory mechanics and pulmonary vascular resistance, thereby reducing perioperative risk; and (3) staged surgical intervention, coupled with multidisciplinary critical-care support, can restore normal cardiopulmonary physiology and permit discontinuation of pulmonary vasodilators within the first year of life.

## Introduction

Congenital pulmonary airway malformations (CPAMs) are the most common congenital thoracic malformations, representing roughly one-quarter of prenatally detected lung lesions and occurring in an estimated 1 per 25,000-35,000 live births [1]. Routine second-trimester ultrasonography and fetal MRI have markedly increased antenatal detection, allowing early risk stratification based on lesion size and morphology [1].

Histologically, CPAMs encompass five Stocker subtypes that reflect aberrant bronchopulmonary development; type 1 lesions - characterized by large, mucous-cell-lined cysts - are both the most prevalent and the variant most strongly linked to later malignant transformation [2].

While many lesions regress or remain clinically silent, CPAMs can precipitate postnatal respiratory failure via mass effect, tension pneumothorax, or secondary infection; moreover, a systematic review of unresected adult congenital lung malformations documented malignant tumors in over 20% of resected CPAMs, underscoring their carcinogenic potential [3].

Accordingly, contemporary expert consensus supports elective lobectomy for asymptomatic infants once they reach approximately three to six months of age (or ≈5 kg body weight), a window that balances the need for early definitive therapy and preservation of lung growth against perioperative morbidity [4].

## Case presentation

A full-term female infant was delivered at 38 weeks’ gestation to a 28-year-old G2P1 mother via uncomplicated spontaneous vaginal delivery. Antenatal ultrasonography at 20 weeks had demonstrated a right-sided CPAM. The diagnosis was confirmed postnatally by chest radiograph performed upon delivery, which revealed a multicystic lesion in the right middle and lower lobes. The neonate had Apgar scores of 8 and 9 at one and five minutes, respectively, and weighed 3.1 kg. She remained asymptomatic on room air and was discharged home after a two-week observation period. In line with contemporary guidelines for asymptomatic CPAMs, which recommend elective surgical resection between three and six months of age to reduce perioperative risk while allowing for somatic growth, the multidisciplinary fetal medicine and pediatric surgery team planned interval thoracoscopic lobectomy at approximately four months, provided the lesion remained clinically silent.

**Figure 1:**
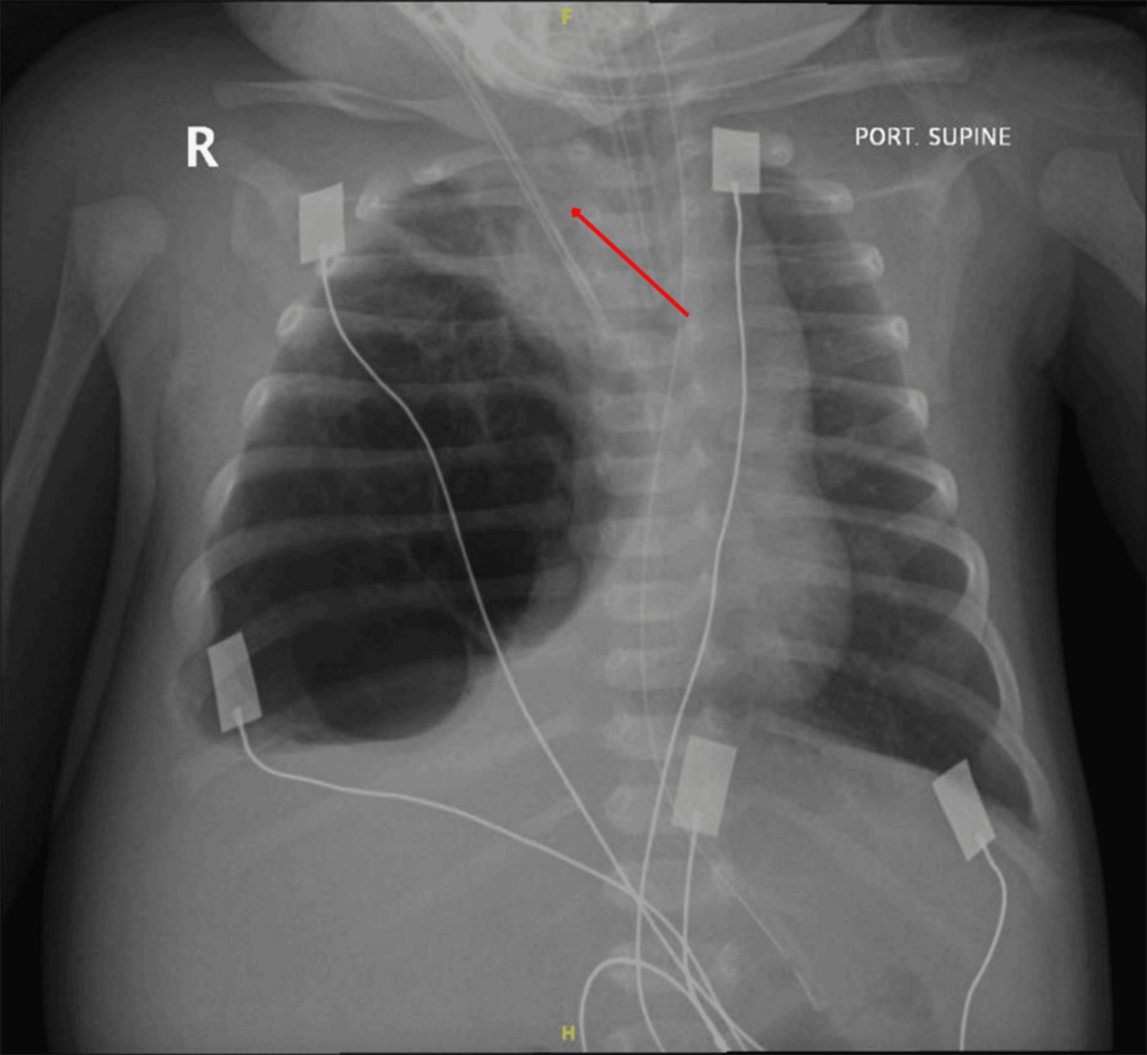
Preoperative anteroposterior chest radiograph at four weeks of age The red arrow indicates a significant mediastinal shift caused by overinflation of the right hemithorax, secondary to the macrocystic CPAM lesion. CPAM, congenital pulmonary airway malformation

**Figure 2:**
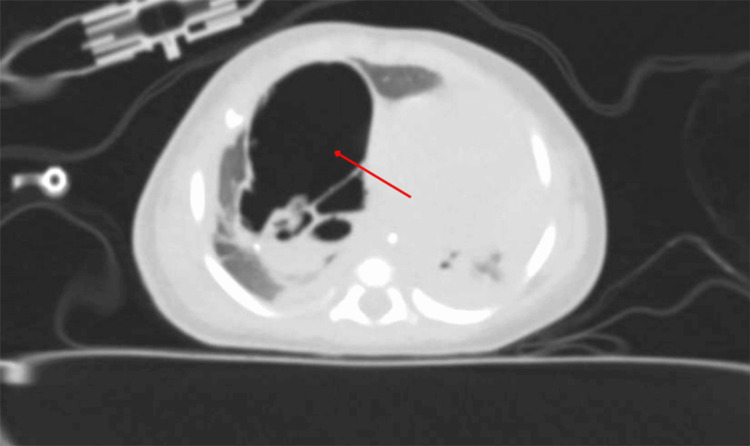
Contrast-enhanced axial chest CT at four weeks of age The red arrow points to a large, air-filled multicystic lesion occupying the right middle and lower lobes, causing displacement of the mediastinum to the contralateral side.

At four weeks of age, she was brought to the emergency department following an abrupt episode of cyanosis and apnea. On arrival, she was hypotonic, bradycardic (heart rate: 60 bpm), and profoundly hypoxemic with an oxygen saturation of 56% on room air. Emergent endotracheal intubation was performed, and she was admitted to the pediatric intensive care unit (PICU). Initial arterial blood gas analysis revealed severe respiratory acidosis (pH: 7.08, PaCO₂: 85 mm Hg). Chest radiography (Figure 1) demonstrated marked hyperinflation of the right hemithorax with contralateral mediastinal shift, and subsequent CT (Figure 2) confirmed enlargement of the prenatally identified cystic lesion, now measuring 7 × 6 × 5 cm.

During the first week of PICU care, the infant experienced recurrent episodes of desaturation and bradycardia, some of which required cardiopulmonary resuscitation. High-frequency oscillatory ventilation (mean airway pressure: 18 cm H₂O) with inhaled nitric oxide at 20 ppm was initiated. On day 6, she developed bilateral tension pneumothoraces requiring emergent tube thoracostomy. Despite aggressive lung recruitment strategies, she remained ventilator dependent with labile oxygen saturations ranging from 70% to 85% and persistent systemic hypotension suggestive of evolving pulmonary hypertension. Sildenafil (1 mg/kg every eight hours) and bosentan (1 mg/kg twice daily) were initiated. Transthoracic echocardiography revealed normal intracardiac anatomy without evidence of fixed elevation in right-sided pressures.

Despite maximal medical therapy - including high-frequency oscillatory ventilation, nitric oxide, and dual pulmonary vasodilator therapy - the infant’s respiratory mechanics and gas exchange continued to deteriorate, and repeated episodes of tension pneumothorax underscored the imminent risk of cardiorespiratory collapse. The planned elective repair was therefore expedited. However, surgery was intentionally delayed until day 42 of life to allow resolution of the acute air leak and optimization of ventilation parameters, further stabilization of pulmonary hypertension under sildenafil and bosentan, completion of preoperative infectious workup with prophylactic antibiotics, and coordination with pediatric anesthesia to secure an appropriately sized double-lumen endotracheal tube.

On the day of surgery, a right posterolateral, muscle-sparing thoracotomy was performed through the fifth intercostal space. Intraoperative inspection demonstrated a multiloculated, balloon-like cyst cluster arising predominantly from the middle lobe and extending into the oblique fissure. The surgical team first decompressed the largest cysts to improve exposure, then performed a stapled wedge resection of the most compromised segment while preserving residual viable parenchyma. Hemostasis was secured with bipolar cautery and topical fibrin sealant, and apical as well as basal 10-Fr chest drains were inserted. Estimated blood loss was 22 mL; the patient remained hemodynamically stable without the need for intraoperative transfusion. Gross examination revealed multiple thin-walled cysts, and detailed histopathological analysis demonstrated extensive hemorrhagic lung parenchyma with clusters of hemosiderin-laden macrophages. Numerous irregularly shaped cystic spaces, ranging from large macrocysts to smaller microcysts, were lined by ciliated columnar epithelium. These cysts were separated by septa that intermittently contained striated skeletal muscle; emphysematous changes and several large-caliber blood vessels were also noted. Collectively, these features were diagnostic of CPAM type 1. Furthermore, trio-based whole-exome sequencing (WES) performed to evaluate for an underlying syndromic association returned negative for pathogenic or likely pathogenic variants. Postoperatively, she developed a pulmonary hypertensive crisis that responded to vasopressor support and augmented inhaled nitric oxide, and she acquired a ventilator-associated pneumonia due to *Stenotrophomonas maltophilia*, successfully treated with intravenous trimethoprim-sulfamethoxazole.

Neurologically, the infant developed multifocal clonic seizures following the initial cardiopulmonary resuscitation early in her PICU course. Levetiracetam was initiated for seizure control. Subsequent brain MRI revealed cortical laminar necrosis, consistent with hypoxic-ischemic injury sustained during the resuscitation event.

Surveillance chest CT and ⁹⁹ᵐTc-macro-aggregated albumin perfusion scan at 11 weeks showed residual dysplastic parenchyma of the right middle lobe with regional perfusion deficits (right lung perfusion 23%). In view of persistent overinflation and the risk of recurrent air leak, definitive completion of middle lobectomy was undertaken at 12 weeks. The procedure was uneventful, and no additional cystic lesions were identified.

**Figure 3:**
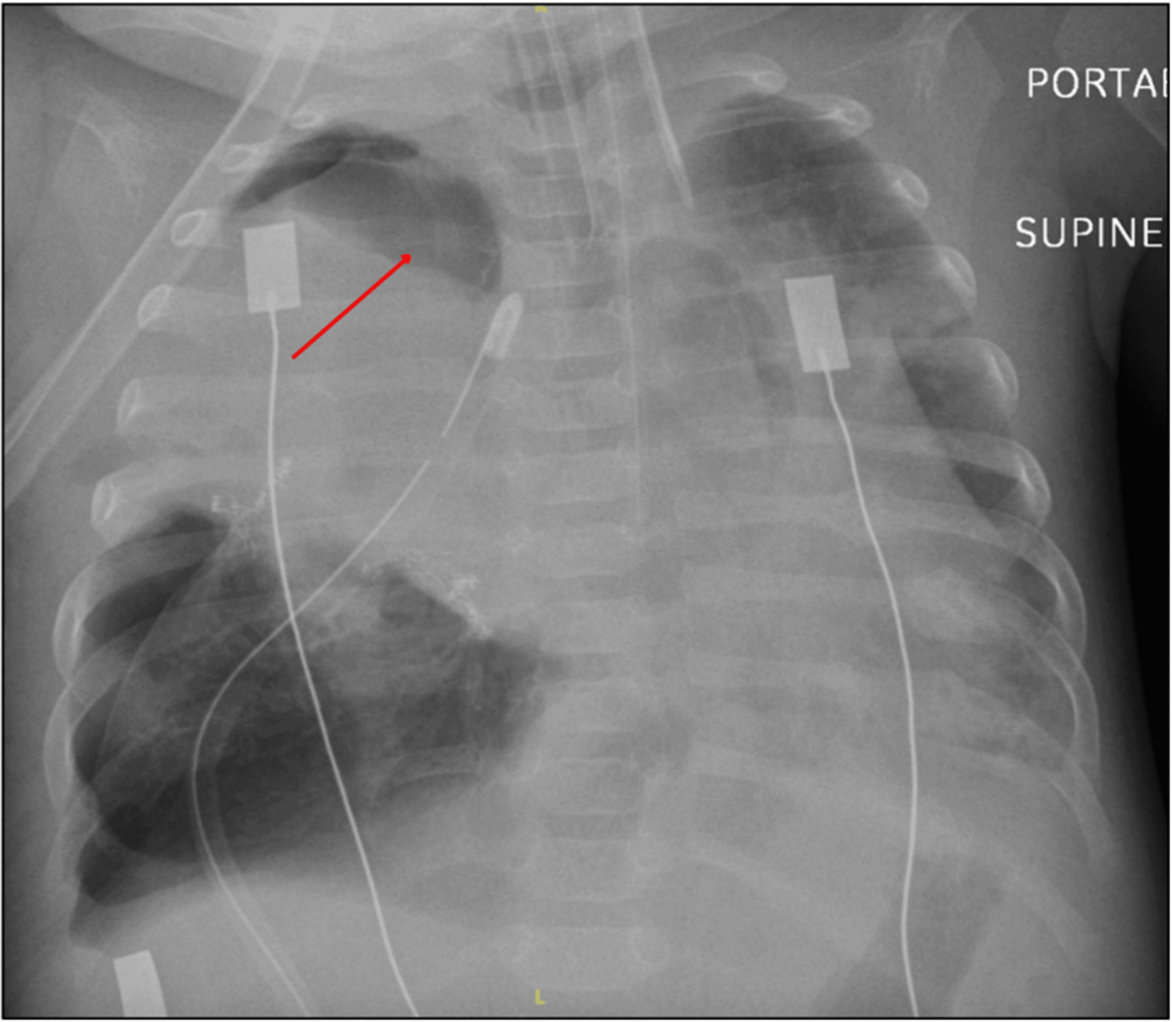
Anteroposterior chest radiograph on postoperative day 3 following right middle lobectomy The red arrow indicates the re-expanded residual right lung with resolution of the mediastinal shift; chest drains remain in situ.

A postoperative anteroposterior chest radiograph obtained on postoperative day 3 (Figure 3) showed re-expansion of the residual right lung with resolution of mediastinal shift and appropriately positioned apical and basal chest drains. Postoperatively, the infant was extubated to nasal bilevel positive airway pressure on day 3; bosentan was tapered during convalescence and discontinued prior to transfer from the PICU, weaned to 0.5 L min⁻¹ low-flow oxygen by day 14, and discharged home at three months of age on nocturnal oxygen. At discharge, she demonstrated appropriate growth, stable cardiorespiratory status, and no new neurological deficits. Follow-up at six months confirmed complete resolution of respiratory symptoms and age-appropriate neurodevelopmental progress. At nine months of age, sildenafil was successfully auto-weaned and discontinued; serial echocardiograms demonstrated normal right-sided pressures, and she has remained off pulmonary vasodilator therapy thereafter.

## Discussion

This neonate’s course highlights the thin line between apparently “silent” CPAMs and life-threatening cardiopulmonary decompensation. Although the lesion was antenatally characterized and initially quiescent, a rapid postnatal increase in cyst volume produced a one-way valve effect, culminating in tension physiology, bilateral air-leak syndromes, and evolving pulmonary hypertension. Such dramatic early deterioration, while uncommon, is well-described: in a recent Thai cohort, 65% of children who ultimately required surgery first became symptomatic in the neonatal period, and those presenting with respiratory distress or air leaks experienced three-fold higher perioperative morbidity than peers whose lesions were electively resected while still asymptomatic [5]. Taken together with our case, these data reinforce that “watchful waiting” must be coupled with clear triggers for escalation and swift access to surgical care.

Several factors pushed the team to delay resection until the sixth postnatal week despite escalating ventilatory support: (1) the need for chest-tube control of bilateral tension pneumothoraces; (2) stabilization of labile pulmonary vascular resistance; and (3) optimization of ventilator settings to permit single-lung ventilation during thoracotomy. Emerging literature supports such short intentional delays: Bhende et al. reported two infants whose acute decompensation was first bridged with percutaneous cyst decompression and ventilation optimization, allowing safer definitive surgery a few weeks later with excellent outcomes [6]. Our staged strategy - initial cyst decompression and wedge resection followed by completion lobectomy - mirrors that paradigm and avoids the hemodynamic swings expected from an immediate formal lobectomy in a patient still on high-frequency oscillation and nitric oxide.

Hypoxia, hyperinflation, and repeated barotrauma triggered pulmonary vasoconstriction and right-to-left shunting. We employed combined sildenafil and bosentan after nitric oxide alone proved insufficient. A recent randomized trial of persistent pulmonary hypertension of the newborn demonstrated that sildenafil achieved a 25% fall in pulmonary artery pressure almost three times faster than bosentan, but that combination therapy was safe and facilitated weaning from inhaled vasodilators [7]. The stepwise wean and eventual discontinuation of both agents in our patient align with those findings and underscore the reversibility of pressure overload once the mechanical culprit is removed.

Tension pneumothorax complicated by contralateral mediastinal shift, as occurred twice in this infant, is a recognized but still underappreciated hazard in macrocystic CPAMs. Misinterpreting the initial radiograph as “simple pneumothorax” may delay definitive therapy; practitioners must therefore maintain a high index of suspicion when a neonate has a known cystic lesion and rapidly progressive respiratory compromise. Contemporary surgical series recommend low-threshold CT imaging in such scenarios to delineate cyst anatomy before chest-tube placement to prevent inadvertent lung injury [6].

Prolonged invasive ventilation and broad-spectrum antimicrobials predisposed our patient to ventilator-associated pneumonia with *S. maltophilia*. Although this organism remains uncommon, it is increasingly reported in neonates receiving quinolone prophylaxis and high-frequency ventilation; targeted therapy with trimethoprim-sulfamethoxazole, as used here, is effective in >90% of pediatric isolates [5].

The hypoxic-ischemic seizures observed postoperatively raise legitimate concern about later neurocognitive function. Reassuringly, a Dutch longitudinal study of children who underwent lung-malformation resection found that, apart from subtle attention deficits at school age, global IQ, memory, and executive function were within population norms [8]. Our patient’s normal developmental milestones at six months echo those data, though ongoing surveillance remains prudent.

Histology confirmed a type 1 CPAM with mucous-cell-lined cysts, precisely the subtype most frequently associated with mucinous adenocarcinoma and pleuropulmonary blastoma in later childhood [2,3]. The recently published Cureus case of invasive mucinous adenocarcinoma arising in a term neonate with CPAM underscores that risk even at the extremes of age [6]. Although trio-based WES was negative in our infant, the result helps exclude recognized cancer-predisposition syndromes and reinforces that somatic transformation, not germline mutation, is the usual pathway to malignancy in CPAM.

## Conclusions

This case demonstrates effective management of a complex CPAM and underscores the importance of prenatal diagnosis, prompt complication recognition, and a collaborative multidisciplinary approach. It also highlights the need for long-term follow-up to address potential neurological outcomes in severe cases. Continued research is required to refine management protocols for complicated CPAM patients.
